# Kinetics of DNA methylation inheritance by the Dnmt1-including complexes during the cell cycle

**DOI:** 10.1186/1747-1028-7-5

**Published:** 2012-02-20

**Authors:** Eric Hervouet, Arulraj Nadaradjane, Marine Gueguen, François M Vallette, Pierre-François Cartron

**Affiliations:** 1 Institut de Recherche Thérapeutique de l'Université de Nantes, INSERM U892, Centre de Recherche en Cancérologie Nantes-Angers, Equipe Apoptose et Progression Tumorale. 8 quai moncousu, BP7021, 44007 Nantes, France; 2 Université de Nantes, Faculté de Médecine, Département de Recherche en Cancérologie, IFR26, F-4400, Nantes, France

**Keywords:** Epigenetic, DNA methylation, Dnmt1, cell cycle

## Abstract

**Background:**

The clonal transmission of lineage-specific DNA methylation patterns in a mammalian genome during the cellular division is a crucial biological process controlled by the DNA methyltransferase Dnmt1, mainly. To investigate possible dynamic mechanisms of DNA methylation inheritance during the cell cycle, we used a Proximity Ligation *In Situ *Assay (P-LISA) to analyze the kinetic of formation and DNA recruitment of Dnmt1-including complexes.

**Results:**

P-LISA, sequential chromatin immunoprecipitation and quantitative methylation specific PCR revealed that the Dnmt1/PCNA/UHRF1-including complexes are mainly formed and recruited on DNA during the S-phase of cell cycle, while the formation and the DNA recruitment of several Dnmt1/transcription factors-including complexes are not S-phase dependent but are G0/G1 and/or G2/M phases dependent.

**Conclusion:**

Our data confirm that DNA methylation inheritance occurs in S-phase, and demonstrate that DNA methylation inheritance can also occur in G0/G1 and G2/M phases of the cell cycle.

## Background

DNA methylation playing a crucial role in the regulation of gene transcription, genomic imprinting, genomic stability, and × chromosome inactivation, its inheritance is essential for the cellular biology and viability because aberrant DNA methylation and targeted disruption of DNA methyltransferase enzymes result in tumorigenicity, lethality or mitotic catastrophe [1-5]. We and others have recently demonstrated that the majority of genomic methylation inheritance is catalyzed by the Dnmt1/PCNA/UHRF1-including complex since its disruption promote global DNA hypomethylation [4,6-8]. Nevertheless, other Dnmt-including complexes can catalyze the genomic DNA methylation inheritance. More generally, the majority of Dnmt1-including complexes are implicated in the inheritance of DNA methylation since Dnmt1 is responsible for maintaining genomic methylation, even if other Dnmt-including complexes could catalyzed maintenance DNA methylation reactions [9,10].

Recently, we reported that the Dnmt1/transcription factors-including complexes act as an alternative mechanism of DNA methylation inheritance to the mechanism performed by the Dnmt1/PCNA/UHRF1-including complex [11]. To complement this point, we here investigated the dynamic of formation of several of these complexes during the cell cycle. Proximity Ligation *In Situ *Assay (P-LISA) and ApoTome technology confirmed that the Dnmt1/PCNA/UHRF1-including complex is mainly formed and recruited on DNA during the S-phase of cell cycle, while the formation and the recruitment on DNA of the six considered Dnmt1/transcription factors-including complexes are not S-phase dependent obligatory. In addition and more particularly, sequential chromatin immunoprecipitation experiments (reChIP) and quantitative methylation specific PCR (qMSP) revealed that Dnmt1/UHRF1 mainly promoted the methylation of the *caspase4 *gene during the S phase, that Dnmt1/YY1 mainly promoted the methylation of the *DR5 *gene during the G2/M phase, and that Dnmt1/Ets1 mainly promoted the methylation of the *caspase1 *gene during the G0/G1 phase.

## Results

### Characterization of cell cycle arrest

To analyze the formation and the recruitment on DNA of the considered Dnmt1-including complexes, U251 cells were blocked into the different cell cycle phases by using serum deprivation and the thymidine, nocodazole or taxol treatments. As expected, cell cycle phase analysis indicated that serum deprivation mainly blocked cells in G0/G1 phases, thymidine treatment mainly blocked cells in S phase nocodazole treatment mainly blocked cells in G2/M phase, while taxol treatment mainly blocked cells in G2/M phase and more specifically in M phase since taxol blocks metaphasis/anaphasis transition of mitosis (Table 1) [12].

**Table 1:** Characterization of cell cycle arrest.

	**Serum dep**	**Thymidine**	**Noco**	**Taxol**
	
**G0/G1**	**93.2 ± 4.7**	10.2 ± 3.7	9.8 ± 4.7	0.2 ± 0.3
**S**	2.6 ± 0.8	**59.7 ± 2.7**	10.8 ± 3.7	2.8 ± 1.7
**G2/M**	2.6 ± 1.7	30.2 ± 3.5	**78.9 ± 3.5**	**81.2 ± 4.1**

U251 cells were treated by serum deprivation (72 h) or by thymidine (2 mM, 24 h), nocodazole (100 ng/mL, 24 h) or taxol (150 ng/mL, 24 h) previous to perform cell cycle analysis by using the NucleoCounter NC-3000™ Kit, Chemometec, France). Data illustrate the mean and standard deviation obtained from 3 independent experiments.

### Dynamic of the Dnmt1-including complexes into cell cycle

**Figure 1:**
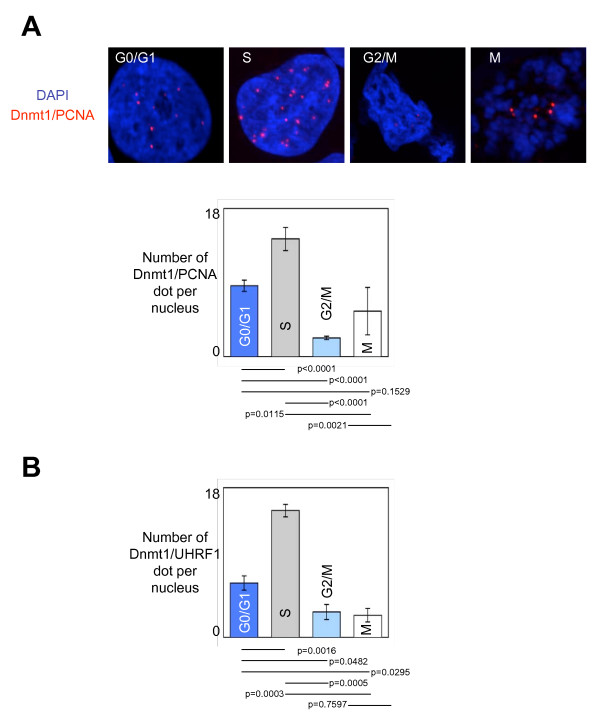
**Dynamic of the formation and of the recruitment on DNA of the Dnmt1/PCNA-including complexes during the cell cycle**. (A) ApoTome view of the Dnmt1/PCNA dots in U251 cells during the different phases of the cell cycle. Red dots symbolize Dnmt1/PCNA dots. Nucleus/DNA are stained in blue via the use of DAPI. Graph (mean ± SEM) illustrating the number of Dnmt1/PCNA dots per nucleus in U251 cells during the different phases of the cell cycle. The number of Dnmt1/PCNA interactions is calculated from the analysis of, at least, 50 nucleus in three independent experimentations. (B) Graph (mean ± SEM) illustrating the number of Dnmt1/UHRF1 interactions per nucleus in U251 cells during the different phases of the cell cycle. The number of Dnmt1/UHRF1 interactions is calculated from the analysis of, at least, 50 nucleus in three independent experimentations.

As already used to analyze the formation of the Dnmt1/PCNA-including complexes and its recruitment on DNA in glioma cells, we used Proximity Ligation *In Situ *Assay (P-LISA) and ApoTome technology to dissect the dynamic of formation of the Dnmt1/PCNA-including complex and its recruitment on DNA during the different phases of cell cycle [4]. Thus, we noted that the Dnmt1/PCNA-including and Dnmt1/UHRF1-including complexes were mainly formed and recruited on DNA during the S-phase of cell cycle (Figures 1A and 1B).

**Figure 2:**
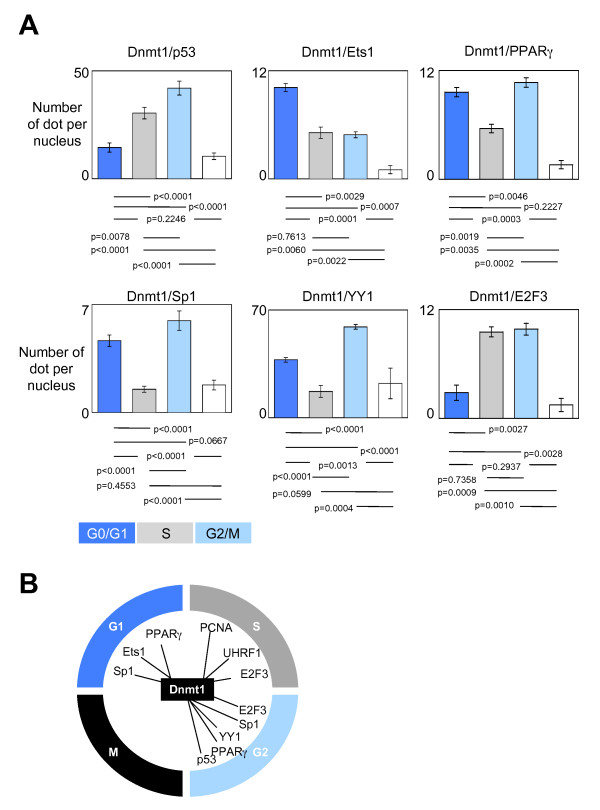
**Dynamic of the formation and of the recruitment on DNA of the Dnmt1/transcription factor-including complexes during the cell cycle**. (A) Graph (mean ± SEM) illustrating the number of Dnmt1/transcription factor dots per nucleus in U251 cells during the different phases of the cell cycle. The number of Dnmt1/transcription factor interactions is calculated from the analysis of, at least, 50 nucleus in three independent experimentations. (B) Schematic representation of the dynamic of the Dnmt1-including complexes during the cell cycle.

Among the transcription factors already described as been potential partners of interactions of Dnmt1, we focused our analysis on the dynamic of formation and recruitment on DNA of 6 Dnmt1/transcription factors-including complexes. P-LISA experiments indicated that the Dnmt1/p53-including and Dnmt1/YY1-including complexes were mainly formed and recruited on DNA during the G2/M phases of cell cycle (Figure 2A). The Dnmt1/Sp1-including and Dnmt1/PPARγ-including complexes were mainly formed and recruited on DNA during the G0/G1 and G2/M phases of cell cycle. The Dnmt1/Ets1-including complexes were mainly formed and recruited on DNA during the G0/G1 phase of cell cycle, while the Dnmt1/E2F3-including complexes were mainly formed and recruited on DNA during the S and G2/M phases of cell cycle. Interestingly, all these data indicated that the formation and the recruitment on DNA of the complex(es) including the Dnmt1, PCNA and/or UHRF1 proteins mainly occur during the S phase of the cell cycle, while the formation and the recruitment on DNA of the Dnmt1/transcription factor-including complexes are not specific of one phase of the cell cycle (Figure 2B).

### Dynamic of the Dnmt1-including complexes and proteins expression during the different cell cycle phases

**Figure 3:**
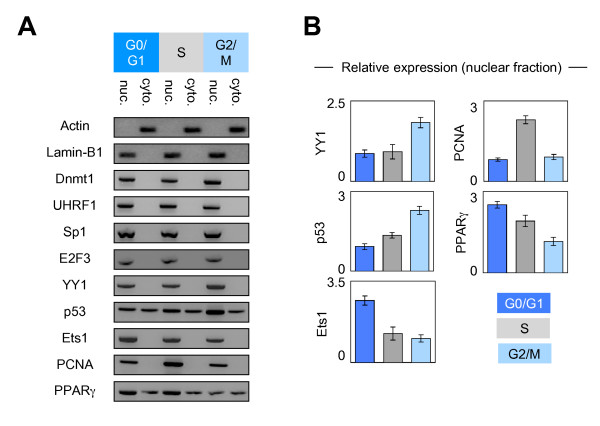
**Dynamic of expression and subcellular localization of the Dnmt1 and its partners of interaction during the cell cycle**. (A) Pictures illustrate the results obtained after Western blot analyses. Nuclear (nuc.) and cytosolic (cyto.) were obtained by using the Nuclear Extract Kit (Active Motif, France). 30 μg and 50 μg of nuclear and cytosolic extracts were used for the western blot analysis concerning the Dnmt1, UHRF1, Sp1, E2F3, YY1, p53, Ets1, PCNA and PPARγ proteins. 20 μg of nuclear and cytosolic extracts were used for the western blot analysis concerning the actin and lamina-B1 proteins. (B) Graphs illustrate the relative expression of indicated proteins in nuclear fraction i.e. the results obtained from the densitometric analyses of western blot (Fusion Fx7 Imager, Fisher Scientific, France and ImageJ software). Lamina-B1 was used as internal control.

Next, we hypothesized that the kinetics of the interactions existing between Dnmt1 and its considered partners could be due to the putative changes in the expression level and/or of subcellular localization of these proteins. To validate these points, we performed western blots analyses using nuclear and cytosolic extracts from cells blocked in the different cell cycle phases. Actin and Lamin-B1 were used as cytosol and nuclear internal controls, respectively. No change in the expression and subcellular localization was observed for Dnmt1, UHRF1, Sp1 and E2F3 (Figures 3A and 3B). YY1 expression was higher in G2/M phase supporting the idea that the Dnmt1/YY1 interactions mainly occur in G2/M phase. Ets1 expression was higher in G0/G1 phase supporting the fact that Dnmt1/Ets1 interaction mainly occurs in G0/G1 phase. PCNA expression was higher in S phase supporting the fact that Dnmt1/PCNA interaction mainly occurs in S phase. With regards to p53, we noted that the p53 expression increased during the cell cycle progression. This observation can explain why Dnmt1/p53 interaction was higher in G2/M phase. Western blot analyses also revealed that the nuclear expression of PPARγ decreased during the cell cycle progression. This point supports the fact that the number of the Dnmt1/PPARγ dots seen in G0/G1 phase was superior to that seen in S phase, but not the observation that the number of the Dnmt1/PPARγ dots seen in S phase was superior to that seen in G2/M phase. Without explaining all variations of Dnmt1/partner-X interactions, the variations in the expression level of the partners of Dnmt1 appears as a rational explanation for the dynamic of the Dnmt1/partner-X interactions existing during the different cell cycle phases.

### Dnmt1/UHRF1, Dnmt1/YY1 and Dnmt1/Ets1 complexes are mainly recruited on genes during the S, G2/M and G0/G1 phases, respectively

**Figure 4:**
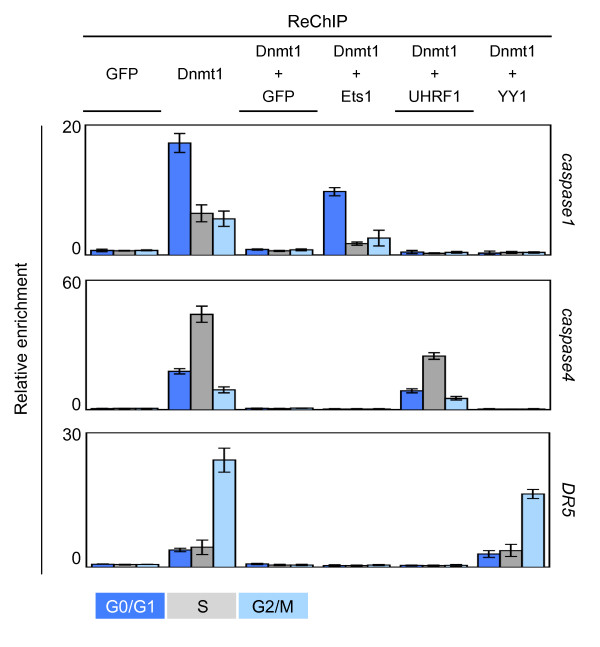
**ReChiP experiments analysis the recruitment on the caspase1, *caspase4*, and *DR5 *genes of the Dnmt1/Ets1, Dnmt1/UHRF1, and complexes during the cell cycle**. Sequential chromatin ImmunoPrecipitation (reChIP) are used to analyze the recruitment of Dnmt1, UHRF1 and transcription factor on the considered genes. Results are expressed as enrichment relative to input and corrected for GFP control levels. The results are averages of at least three independent experiments with error bars indicating standard deviation.

Consistent with our last results, we then hypothesized that the complexes including the Dnmt1/Ets1, Dnmt1/UHRF1 and Dnmt1/YY1 proteins were mainly recruited on DNA during the G0/G1, S or G2/M phases, respectively. To cure these points, we analyzed by sequential chromatin immunoprecipitation (reChIP) experimentations the co-recruitment of Dnmt1/Ets1, Dnmt1/UHRF1 and Dnmt1/YY1 on specific genes during the different phases of the cell cycle. Thus, after a blockade of cells in the different cell cycle phases, chromatin was immunoprecipitated with an antibody directed against Dnmt1 in the first time and with an antibody directed against Ets1, UHRF1 or YY1 in a second time, an antibody directed against GFP was used as control. For the targeted genes, we focused our analyses on genes coding for effectors or regulators of apoptosis since our group is involved in the analysis of mechanisms modulating apoptosis. As such, we analyzed the co-recruitment of Dnmt1/Ets1, Dnmt1/UHRF1 and Dnmt1/YY1 on *caspase-1 *gene (a target of Ets1), *caspase-4 *gene and *DR5 *gene (a target of YY1) [13,14]. As shown in Figure 4, we observed that the Dnmt1/UHRF1 complex was mainly recruited on the *caspase1 *gene in G0/G1 phase and that the recruitment of Dnmt1/UHRF1 and Dnmt1/YY1 on the *caspase4 *and *DR5 *gene mainly occurred during the S and G2/M phases, respectively. Associated with our previous data, these last results strongly support the idea that the Dnmt1/UHRF1, Dnmt1/YY1 and Dnmt1/Ets1 complexes were mainly formed and recruited on DNA during the S, G2/M and G0/G1 phases of the cell cycle, respectively.

### Maintenance of DNA methylation can occur after cellular division

**Figure 5:**
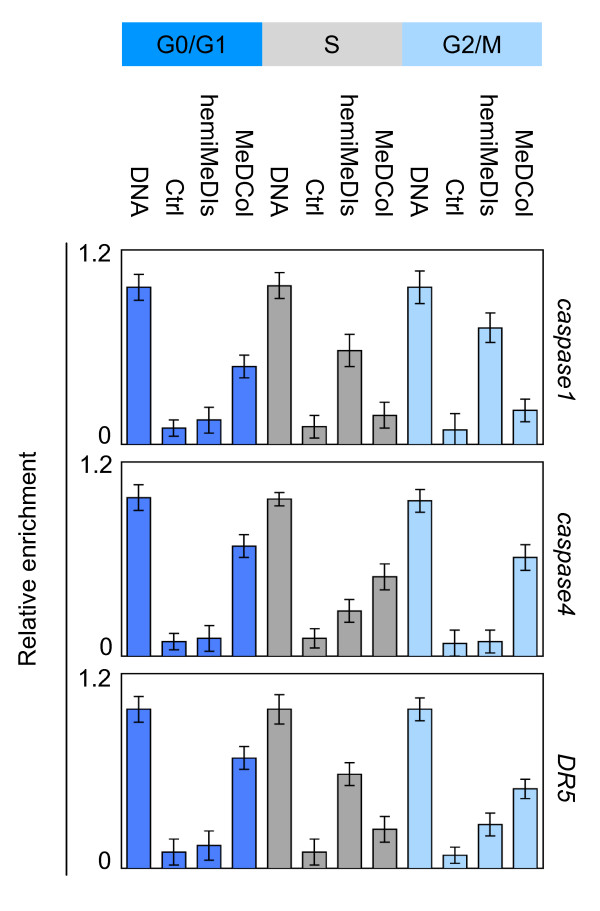
**Methylation status of the *caspase1, caspase4 *and *DR5 *during the cell cycle**. DNA: sonicated DNA (input), Ctrl: hemiMeDIs realized in absence of His-UHRF1 on column, hemiMeDIs: hemi-methylated DNA Isolated, MeDCol: Methylated DNA collected. The results are averages of at least three independent experiments with error bars indicating standard deviation.

The fact that the formation and the recruitment on DNA of the Dnmt1/Partner-X occurs in different phases suggests that the inheritance of DNA methylation on its targeted genes by this type of complex can occur after the cellular division or that DNA can exist under a hemi-methylated status in one or several cell cycle phases. Thus, we compared, the methylation status of *caspase1, caspase4 *and *DR5 *genes during the different cell cycle phases. Methylated DNA Collected (MeDCol) and hemi-Methylated DNA Isolated (hemi-MeDIs) were used to determine the status of methylation or hemi-methylation of the considered genes since DNA was purified using recombinant proteins having the capacity to specifically bind methylated (MBD2b/MBD3L1 protein complex, according to Active Motif kit) or hemi-methylated DNA (UHRF1). The hemi-MeDIs and MeDCol indicated that *caspase1 *gene was mainly hemi-methylated in S and G2/M phases and was mainly methylated in G0/G1 phase since maximum of amplification of *caspase1 *gene was observed when PCR were realized from DNA isolated by hemiMeDIs and MeDCol issue to cells blocked in S and G2/M, and G0/G1 phases, respectively (Figure 5). Similar experiments indicated that caspase4 gene was hemi-methylated in S phase and methylated in G0/G1 and G2/M phases, and DR5 was hemi-methylated in S phase and methylated in G0/G1 phase (Figure 5).

Consistently with results obtained from reChIP experiments, our data indicated that the maintenance of methylation of *capsase1 *gene occurs during G0/G1 phase and implicates Dnmt1/Ets1, the maintenance of methylation of *capsase4 *gene occurs during S phase and implicates Dnmt1/UHRF1, and that the maintenance of methylation of *DR5 *gene occurs during G2/M phase and implicates Dnmt1/YY1.

## Discussion

During the last decades, several studies have analyzed the crosstalk existing between the mechanism of DNA methylation and cell cycle since DNA replication, occurring during S phase, gives birth to a hemi-methylated DNA of which the neo-synthesized strand must be methylated to assure the transmission of the DNA methylation during the cell division. Thus, supported by this idea, by the preferential activity of Dnmt1 for hemi-methylated DNA, and by the description of the interaction between Dnmt1, PCNA (a protein of the DNA replication machinery) and UHRF1 (a protein binding hemi-methylation DNA), it has been proposed that the inheritance of DNA methylation by the Dnmt1/PCNA/UHRF1-including complex occurs during the S phase of cell cycle [6,8,15,16]. However, the idea that the DNA methylation occurs during S phase, although confirmed by several publications, is challenged and discussed by the fact that the maintaining of DNA methylation could be catalyzed in other phases of the cell cycle. Besides, our data echoes this situation since we observed that the Dnmt1/PCNA-including complexes were mainly formed and recruited on DNA during the S phase of cell cycle, while the formation and the DNA recruitment of the Dnmt1/transcription factor-including complexes can occur during different phases of the cell cycle. The fact that the formation and the recruitment on DNA of some Dnmt1/transcription factor-including complexes (such as the Dnmt1/p53-including or Dnmt1/YY1-including complexes) mainly occur during G2/M phases confirms the idea that Dnmt1 can load chromatin during the G2 and M phases [17]. Consistently with this point and with the fact that the maintenance of DNA methylation pattern of certain genes can be catalyze by the Dnmt1/transcription factor complexes, it appears that the inheritance of DNA methylation mainly requires Dnmt1 but not its interaction with the replication machinery such as already reported in the literature [11,18]. Moreover, the dynamism of the Dnmt1/PCNA interaction during the cell cycle also reinforces the transient nature of this interaction [19]. Similarly, the dynamism of the considered Dnmt1/transcription factor interactions suggests that these interactions are also transient. Thus, the description of dynamic mechanisms of DNA methylation inheritance during the cell cycle by the considered Dnmt1-including complexes increases our understanding of the orchestration of different Dnmt-including complexes into the process of DNA methylation inheritance. More particularly, after the work published by Métivier et al., our work completes and provides new perspectives into the investigation of the cyclical DNA methylation process [20].

The observation that the interaction level of Dnmt1 with certain of its interaction partner is parallel to the expression level of this partner, provides a rational explanation for the existence a dynamic for Dnmt1/partner-X interaction occurring during the cell cycle. Concerning the kinetics of expression of the PCNA, YY1, p53, Ets1 and PPARγ proteins, our data revealed the existent of significant but weak variations (2.8 fold for the maximum). The elevated expression of PCNA in S phase seems make sense with its function during cell cycle phase. The elevated expression of Ets1 in G0/G1 phase and of p53 and YY1 in G2/M phase appears consistent with the description of their activation/function during these cell cycle phases [21-24]. Concerning the impact of nocodazole on p53, our data indicate that elevation of p53 expression was not associated with a nocodazole-induced up-expression of p53 since the presence of cycloheximide (an inhibitor of protein synthesis) not changes the p53 expression (Additional file 1).

The variation of the protein expression does not explain all the dynamics of the Dnmt1/partner-X interactions occurring during the cell cycle. A description of other causes of the dynamic of Dnmt1/partner-X interaction occurring during the cell cycle is an ongoing subject in our laboratory. We have also the project to develop a global analysis identifying a maximum of genes of which the methylation inheritance occurs in the different cell cycle phases. Nevertheless, despite its limitation to low numbers of genes and on Dnmt1-including complex taken into consideration, our data provide an interesting proof of concept of the kinetics of DNA methylation inheritance by the Dnmt1-including complexes during the cell cycle.

The fact that the formation and the recruitment on DNA of some Dnmt1/transcription factor-including complexes such as the Dnmt1/Ets1-including complexes mainly occur in G0/G1 phases suggests that the inheritance of DNA methylation on the targeted genes by this type of complex can occur after the cellular division. In other terms, this point suggests that the cellular division can occur with a significant and certain stock of hemi-methylated DNA i.e. that the cellular division can occur in absence of total inheritance of DNA methylation hallmark characterizing the "mother cell". Besides, our data illustrate this point by showing that the Dnmt1/Ets1 complex promotes the maintenance of the *caspase1 *gene in G0/G1 phases i.e. after the cellular division.

Finally, the identification of the Dnmt1/Ets1, Dnmt1/UHRF1 and Dnmt1/YY1 as actors of the DNA methylation of the *caspase1, caspase4 *and *DR5 *genes respectively reinforces our understanding of the epigenetic regulation of genes coding for proteins playing a role of actor or regulator of the apoptotic program [25]. Besides, the crucial role played by the Dnmt1 into these processes is also supported by the fact that the Dnmt1 invalidation abrogated the methylation of the *caspase1, caspase4 *and *DR5 *genes [26]. In addition, the identification of the Dnmt1 as a crucial actor of the methylation of the *caspase1, caspase4 *and *DR5 *genes suggests that the use of Dnmt1 inhibitor such as the MG-98 (a second-generation Dnmt1-specific anti-sense inhibitor currently in phase II clinical trials) or the SGI-1027 could be used to promote or restore the sensitivity of apoptosis induced by the TNF or TRAIL systems in glioma [27,28].

## Conclusion

Our data confirm that DNA methylation inheritance occurs in S-phase, and clearly demonstrate that DNA methylation inheritance can also occur in G0/G1 and G2/M phases of the cell cycle. Thus, and despite its limitation to low number of genes and on Dnmt1-including complex taken into consideration, our data provide, for the first time, an interesting proof of concept of the kinetics of DNA methylation inheritance by the Dnmt1-including complexes during the cell cycle.

## Methods

### Proximity ligation in situ assay (P-LISA)

Cells were fixed with 4% paraformaldehyde in PBS pH7.4 for 15 min at room temperature. Permeabilization is performed with PBS containing 0.5% Triton 100 × 4 for 20 min at room temperature and staining were realized according to manufacturer's instructions (Olink Bioscience) [29]. Fluorescence was visualized by using the Axiovert 200 M microscopy system (Zeiss, Le Pecq, France) with ApoTome module (X63 and numerial aperture 1.4). Pictures acquisition was realized in structured illumination microscopy [30]. Thus, the lateral resolution (rl) is, according to the Rayleigt criteria: rl = 0.61λ/NA (λ: wavelength; NA: numerical aperture of the objective), and the axial resolution, in ApoTome, is defined by the full-width half maximum (FWHMz)

$$FWHM\left(z\right)=\frac{3.83}{16\pi }\cdot \frac{\lambda \times 10^{-3}}{n\cdot \sin^{2}\left(\alpha \;/)\;2\right)\bar{v}\left(1-\bar{v}\;/)\;2\right)}$$

In this equation, λ is the emission wavelength, n is the index of medium refraction, ν is the frequency and α is the angle of opening of the objective as previously described [31].

After decovolving (3.5 Huygens Essential software (SVI)), 3D view was obtained by using Amira.4.1.1 program. Finally, the images were analyzed by using the freeware "BlobFinder available for download from http://www.cb.uu.se/~amin/BlobFinder. Thus, we obtained either number of signals per nuclei since nuclei can be automatically identified. In other terms, the use of this program participates to the normalization, standardization, reproducibility and to the definition of the cut off signal to accept/quantify or not a dot.

### Chromatin ImmunoPrecipitation (ChIP) and Re-ChIP experiments

Briefly, chromatin was purified from cells after cross-linking with 1% formaldehyde for 10 min at room temperature. ChIP and Re-ChIP assays were performed with the ChIP-IT™ and Re-ChIP-IT™ kit (ActiveMotif, France) with indicated antibodies and primers. In ChIP and reChIP assays, quantitative PCRs (MX4000 system and the Brilliant SYBR Green QPCR Core Reagent Kit) were performed on 2 μl of input, ChIP or reChIP sample DNA. The relative levels of the fragments of interest in the immunoprecipitated DNA were determined from the threshold cycle (*CT*) for each PCR. To ensure the reliability of our ChIP and reChIP data, two control samples specific for the ChIP and reChIP experiments have been included: the input sample (indicative of the presence and amount of chromatin used in the ChIP reaction) and the control antibody (GFP antibody) sample (indicative of the amount of background signal generated by the chromatin preparations and ChIP procedure). The calculations of the relative enrichment values were as described below. (i) We normalized the quantitative PCR signals obtained from the immunoprecipitated ChIP sample to the input sample, i.e., *CT *input - *CT *ChIP. The PCR efficiency, corresponding to the different sets of primers used in our quantitative PCR, was then raised to the power of this *CT *difference, i.e., (primer PCR efficiency)(*CT *input - *CT *ChIP). (ii) The enrichment (*n*-fold) of the immunoprecipitated sequence of interest was obtained by normalizing the values to the ChIP background (relative to IP GFP antibody). (iii) To ensure that the observed binding of the tested proteins reflect specific binding to the *considered *promoter, we also amplified an unrelated control region in a quantitative PCR. The relative enrichment values were calculated by dividing the enrichment (*n*-fold) derived from the sequence of interest by the signal derived from this control locus (unrelated control region).

### DNA extraction and methylation status analyses

DNA was extracted by using the QiaAmp DNA mini Kit (Qiagen, France). DNA was sonicated by using a Bioruptor Sonicator (Diagenode, France). Methylated DNA Collected (MeDCol) was realized by using the MethylCollector™ Ultra kit (Active Motif, France). HemiMethylated DNA Isolated (hemiMeDIs) was realized by using his-tagged UHRF1 protein to isolated hemimethylated DNA. Briefly, 2 μg of His-UHRF1 were loading on Handee™ Spin Column containing 50 μl of Immobilized Cobalt Chelate resin (ProFound™ Pull-Down PolyHis Protein:Protein Interaction Kit Pierce, Thermo Scientific, France). Next, 100 ng of sonicated genomic DNA was incubated on previous tube/column, on a rotisserie shaker for 1 hour at 4°C in binding buffer issue to the MethylCollector™ Ultra kit (Active Motif, France). Washes, recovery of methylated DNA fragments, DNA clean-up steps were performed such as described in MethylCollector™ Ultra kit (Active Motif, France). Finally, methylated and hemi-methylated DNA was analyzed by qPCR. To evaluate the relative enrichment of target sequences, we normalized (for each amplicon tested) the C_t _of the MeDCOl/HemiMeDIs fraction to the C_t _of the input (ΔC_t_). Subsequently we normalized the ΔC_t _of each target sequence to the ΔC_t _of an unmethylated control sequence (ΔΔC_t_). Finally, we calculated the relative enrichment E = 2^ΔΔCt^.

### Western blot

In brief, proteins were size fractionated by sodium dodecyl sulfate-polyacrylamide gel electrophoresis. Proteins were transferred onto nitrocellulose or PVDF membrane. Saturation and blotting were realized by using SNAP i.d™ Protein Detection System (Millipore, France). The detection of proteins was performed using ECL™(Amersham Biosciences) and/or SuperSignal west femto Maximum Sensitivity (Pierce) chemilumenscence reagents.

### Supplemental data

Antibodies and primers are listed in additional file 2 and 3.

## Competing interests

The authors declare that they have no competing interests.

## Authors' contributions

All authors have read and approved the final manuscript. PFC AND FMV designed the experiments, PFC, EH, MG and AN performed all the experiments and data analysis. PFC and FMV wrote the manuscript and are responsible for the scientific contents of the manuscript.

## Supplementary Material

Additional file 1 **List of antibodies**. Click here for file

Additional file 2 **List of primers**. Click here for file

Additional file 3 **Effect of cycloheximide and/or nocodazole treatment on the p53 expression**. Western blot analyses indicated that the cycloheximide treatment unchanged the elevation of p53 expression seen when U251 cells were treated with nocodazole.Thus, we supposed that nocodazole treatment not activated the synthesis of p53 and that the elevation of p53 in nocodazole-treated cells was due to an accumulation of p53. Click here for file
