# Supplementary figures and images for: Kinetics of DNA methylation inheritance by the Dnmt1-including complexes during the cell cycle
Source: Cell Div. 2012 Feb 20;7:5. doi: 10.1186/1747-1028-7-5 (PMC3307489; doi:10.1186/1747-1028-7-5)

Nocodazole (100ng/mL, 24h)      -      +      +

Cycloheximide (20μM, 24h)      -      -      +

Actin

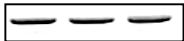

p53

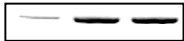

Supplement: Additional file 1 — List of antibodies. [file 1747-1028-7-5-S1.PDF]
