# Supplementary material for: Kinetics of DNA methylation inheritance by the Dnmt1-including complexes during the cell cycle
Source: Cell Div. 2012 Feb 20;7:5. doi: 10.1186/1747-1028-7-5 (PMC3307489; doi:10.1186/1747-1028-7-5)
Supplement: Additional file 2 — List of primers. [file 1747-1028-7-5-S2.PDF]

**Antibodies**	**references**
Dnmt1	Tebu-Bio sc10221
PCNA	Abcam ab18197
UHRF1	Abcam ab57083
Sp1	Tebu-Bio sc59
p53	BD-Pharmingen 554294
PPAR	Tebu-Bio sc7273
YY1	Tebu-Bio sc1703
Ets1	Tebu-Bio sc55581
E2F3	Tebu-Bio sc28308
GFP	Tebu-Bio sc996ac
