# Supplementary material for: Kinetics of DNA methylation inheritance by the Dnmt1-including complexes during the cell cycle
Source: Cell Div. 2012 Feb 20;7:5. doi: 10.1186/1747-1028-7-5 (PMC3307489; doi:10.1186/1747-1028-7-5)
Supplement: Additional file 3 — Effect of cycloheximide and/or nocodazole treatment on the p53 expression. Western blot analyses indicated that the cycloheximide treatment unchanged the elevation of p53 expression seen when U251 cells were treated with nocodazole.Thus, we supposed that nocodazole treatment not activated the synthesis of p53 and that the elevation of p53 in nocodazole-treated cells was due to an accumulation of p53. [file 1747-1028-7-5-S3.PDF]

gene	ChIP
caspase1	S: ACATTCTGAGTCCAGAGCC<br>AS:ATTCTGCACTCAAGCAATTCA
caspase4	S:CCCAGCCTCCCTAGCAC<br>AS:TAGAATCCCCACTAGCC
DR5	S: ACTCCCCAAGTGCCTCC<br>AS: GGCTGTGGTTTGTTTCTGG
